# Supplementary material for: Toblerone: detecting exon deletion events in cancer using RNA-seq
Source: F1000Res. 2023 Feb 3;12:130. [Version 1] doi: 10.12688/f1000research.129490.1 (PMC10521068; doi:10.12688/f1000research.129490.1)
Supplement: Peer review history

# Reviewer report: Reviewer response for version 1

**DOI:** 10.5256/f1000research.142177.r192103

**Published:** 2023-09-07

**Authors:** Matt Field

**Affiliations:** 1 James Cook University, Cairns, Australia

**General comments:**

 Here Lonsdale et al describe Toblerone, software designed to detect exon deletions in known genes from RNA-Seq. Overall this tool is useful for targeted applications and would likely be of interest to the broader research/clinical community. The manuscript is well written and encouragingly all relevant code and supporting documentation is available enabling other research/clinicians to utilise the framework. My comments below are relevant to the specific sections:

**Methods: “Overview”:**

- Define equivalence class.
- While the total counts is a useful measure in some contexts, did you think about comparing/reporting the read count supporting the deletion to only the reads that definitively do not support the deletion? For example in a deletion skipping exon 2, supporting reads would span exon 1-3 while non-support reads would be the sum of exons spanning boundaries 1-2 and 2-3.   

**Methods: Index**

- In some instances, non-canonical forms can be important.  Is it possible to input multiple isoforms for the same gene for indexing?
- Is there any handling for aberrant read alignments that don’t clearly skip canonical exons?  For example supporting reads for a deletions that span a canonical exon boundary and a non-canonical exon boundary might be important to the patient treatment.

**Methods: “app”**

- The app is a great idea and should be useful in targeted cases.   Are there any plans to allow users to request additional applications for other relevant genes?  It would be great to see a bit more documentation in the app README on github.

**Results: ALL**

- Just to clarify, the exon 4 and 6 deletions are not related to ALL?  Might be useful to display the most common non-ALL and ALL-specific isoforms.

**Results: Parameters effect**

- Do deletions being in frame and out of frame have any impact on the algorithm? 
- What, if any impact does the length of each alignment segment have on the call?  For example, is there any difference between one read that has 140 bases aligned to one exon and only 10 bases aligned across the skipped exon whereas another read has 75 bases aligned to each. Similarly is the mapping score and the uniqueness of each alignment segment factored in?

**Results: CD22**

- Nice example of another application.  While the paper largely describes the application for IKZF1, it’s uptake will largely be determined by how generalisable the framework is. For example, determining the cutoffs in Fig 2C and the placement of the new sample relative to others in Fig 3 is specific to IKZF1 but it would be useful to describe what specifically is required for any new proposed use case.
- Do you suspect each application (i.e. gene) will require custom cutoffs/filters?

**Discussion:**

- Do you suspect each application (i.e. gene) will require custom cutoffs/filters?
- A specific application would be identifying somatic events in paired tumour / normal samples. Is there any thought to further developments/optimisations around this common use case?
- I could imagine scenarios where it is desirable to interrogate a small panel of commonly mutated genes specific to type of cancer (similar to targeted gene panel sequencing).  Are there are limitations that might present challenges to this level of scaling?

Is the work clearly and accurately presented and does it cite the current literature?

Yes

If applicable, is the statistical analysis and its interpretation appropriate?

Yes

Are all the source data underlying the results available to ensure full reproducibility?

Yes

Is the study design appropriate and is the work technically sound?

Yes

Are the conclusions drawn adequately supported by the results?

Yes

Are sufficient details of methods and analysis provided to allow replication by others?

Yes

Reviewer Expertise:

bioinformatics

I confirm that I have read this submission and believe that I have an appropriate level of expertise to confirm that it is of an acceptable scientific standard.

# Reviewer report: Reviewer response for version 1

**DOI:** 10.5256/f1000research.142177.r163943

**Published:** 2023-04-25

**Authors:** Katherine Pillman

**Affiliations:** 1 Centre for Cancer Biology, an Alliance between SA Pathology, University of South Australia, Adelaide, Australia

**Article Summary**

 The article describes ‘Toblerone’, an improved targeted method of identifying and quantifying focal deletions of whole internal exons in one or a few genes. The method works without a priori knowledge of which exons will be deleted and was developed to improve the accuracy and sensitivity of detection of exonic deletions for a clinically important gene, IKZF1.

 Overall, I think this is a clearly written and well-explained approach that describes the design and implementation of a solution to the clinically important problem identified in a previous study by the group (Brown et al. 2007). The provided app provides an easy visual way of assessing the results and could also be used as a template when expanding the method to other genes. I think that the approach of creating individual transcripts defining each potential set of exonic deletions is a good one; it deals with some tricky edge cases for mapping reads across splice junctions in a reasonable and transparent way. For example, other solutions wherein one maps reads to only the two junction-flanking exons can raise problems when one of these exons is much shorter than the read length.

**Major comments**

 None.

**Minor comments**

 Including a brief explanation of any of the following terms would make the paper more widely accessible:

- Focal deletion
- Pseudoalignment
- Equivalence Class Level

In the calculation of Scaled Proportion, it seems to me that the trim value should be part of the equation as it affects the proportion of reads that would be expected to be detected over a junction for a given sequencing depth. Might the solution be to subtract the trim length (=5 by default) from the read length prior to calculation, to account for the loss of the use of the last 5 bases? Would it be correct to think that the length of the part of the read available for counting is what the data should be normalised by?

 I could not find figure legends for the Supp Figures, can these please be explicitly described somewhere.

 If it is possible, a simple explanation of the meaning of the Scaled Proportion metric would be a great help to the user intuitively understanding their results. Is it “the fraction of reads across a deletion junction relative to what would be expected for a given expression level and read length”? Meaning it is an estimate of the true fraction of transcripts present in Sample X which contain that deletion, with a theoretical maximum of 1.0 (though only in a perfect world where read coverage is not stochastic or biased across a transcript)?

 On the last line of Page 7, "Supplementary figure 3" should be changed to "Supplementary figure 4".

 In Fig3C, I found it confusing that the purple dashed line represents the single sample position, whereas similar lines in other figures (purple lines in Fig3A, dashed lines in Fig2C) were used to describe the thresholds. I think it would help if this line can replaced with a different coloured symbol and/or changed to a new colour.

 I also thought that if a genome-mapped bam was used as the first step, it is important that the mapper used allows soft clipping (like STAR in default mode). A mapper that does not soft clip might discard reads which had a smallish number of bases mapping across a non-canonical junction, which would reduce the sensitivity of Toblerone. This could be worth noting in the text.

 A suggestion: a great use of Toblerone would be in conjunction with an in-clinic capture panel, with probes tiled on the gene specifically so as to avoid capture bias over exon-exon junctions. The huge sequencing depth one gets from capture panels would solve the issues described in determining whether the samples with 0 counts lack the genomic fusion or simply have low sequencing coverage.

Is the work clearly and accurately presented and does it cite the current literature?

Yes

If applicable, is the statistical analysis and its interpretation appropriate?

Yes

Are all the source data underlying the results available to ensure full reproducibility?

Yes

Is the study design appropriate and is the work technically sound?

Yes

Are the conclusions drawn adequately supported by the results?

Yes

Are sufficient details of methods and analysis provided to allow replication by others?

Yes

Reviewer Expertise:

Transcriptome bioinformatics, gene regulation.

I confirm that I have read this submission and believe that I have an appropriate level of expertise to confirm that it is of an acceptable scientific standard.
